# Ultraviolet light illuminates the avian nature of the Berlin *Archaeopteryx* skeleton

**DOI:** 10.1038/s41598-019-42823-5

**Published:** 2019-04-24

**Authors:** Daniela Schwarz, Martin Kundrát, Helmut Tischlinger, Gareth Dyke, Ryan M. Carney

**Affiliations:** 1 Museum für Naturkunde, Leibniz Institute for Evolution and Biodiversity Science, 10115 Berlin, Germany; 2 Center for Interdisciplinary Biosciences, Technology and Innovation Park, University of Pavol Jozef Šafárik, 04154 Košice, Slovakia; 3 Tannenweg 16, 85134 Stammham, Germany; 4 Department of Geology, Babes-Bolyai University, Cluj-Napoca, Romania; 5 Department of Integrative Biology, University of South Florida, 33620 Tampa, FL USA

**Keywords:** Evolution, Palaeontology

## Abstract

The question of whether the iconic avialan *Archaeopteryx* was capable of active flapping flight or only passive gliding is still unresolved. This study contributes to this debate by reporting on two key aspects of this fossil that are visible under ultraviolet (UV) light. In contrast to previous studies, we show that most of the vertebral column of the Berlin *Archaeopteryx* possesses intraosseous pneumaticity, and that pneumatic structures also extend beyond the anterior thoracic vertebrae in other specimens of *Archaeopteryx*. With a minimum Pneumaticity Index (PI) of 0.39, *Archaeopteryx* had a much more lightweight skeleton than has been previously reported, comprising an air sac-driven respiratory system with the potential for a bird-like, high-performance metabolism. The neural spines of the 16^th^ to 22^nd^ presacral vertebrae in the Berlin *Archaeopteryx* are bridged by interspinal ossifications, and form a rigid notarium-like structure similar to the condition seen in modern birds. This reinforced vertebral column, combined with the extensive development of air sacs, suggests that *Archaeopteryx* was capable of flapping its wings for cursorial and/or aerial locomotion.

## Introduction

Living birds have extensively pneumatized postcrania, due to their unique and extremely efficient respiratory system^1,2^. This adaptation includes a series of pneumatic diverticula that derive from large air sacs, encompass the lungs, and invade different parts of the skeleton^3^, leaving clear marks (pneumatic foramina) found in both avian and non-avian dinosaurs^4–6^. Reconstructions of pneumatic structures in fossil archosaurs have been previously based on comparisons with extant birds^1,2,5–8^. Evidence used to infer intraosseous pneumaticity for dinosaurs, in particular isolated foramina in compact bone or “blind” fossae, remains ambiguous however, as these cavities can house other structures such as muscles, fat, or neurovascular tissue^1^. The only unambiguous, reliable indicators of intraosseous pneumaticity are cortical foramina and communicating fossae connected with larger cavities within the bone^1^. Furthermore, the locations of foramina in the vertebrae or limb bones of dinosaurs that are comparable to those in extant birds support the interpretation of such structures as pneumatic^1,2,7^. The large cavities within pneumatic vertebrae of dinosaurs have been descriptively separated according to their architecture into larger and rounded camerae, and smaller and more angular-walled camellae^8–10^. Both types of internal pneumatic structures can occur in the same skeletal element, and multiple camellae can create a specific honeycomb-like pattern as in extant birds, described as somphospondylous^10^. The anteroposterior extension and spatial distribution of intraosseous pneumaticity provides critical anatomical evidence enabling reconstructions of the respiratory apparatus. The extent of pneumaticity also enables assessment of body weight, locomotor preferences, and metabolic activity, and therefore provides insights on the paleobiology and behavior of extinct animals^5–7^.

In *Archaeopteryx*, both the presence and extent of intraosseous pneumaticity has been controversially discussed ever since the 19th century^11^. A recent microtomographic study on the Daiting specimen (*Archaeopteryx albersdoerferi*) showed that all cranial bones, shoulder girdles, and wing bones contain internal pneumatic cavities^12^. Similar observations have been based on the Berlin, London, and Eichstätt specimens, which preserve foramina on the surfaces of their presacral vertebrae and pubes^1,7,13–15^. Pneumatic foramina have been described in the 2^nd^ to 5^th^ cervical vertebrae^1,7,13,15^ and posterior presacral vertebrae of the Berlin *Archaeopteryx*^16,17^; this specimen is also known to have hollow thoracic ribs^13^. The most unequivocal pneumatic structure seen in the postcranial skeleton of the Berlin specimen is the pneumatic foramen in the body of the 5^th^ cervical vertebra^1^, exemplifying the ‘common pattern’ seen in earlier-diverging theropod dinosaurs^7^.

**Figure 1:**
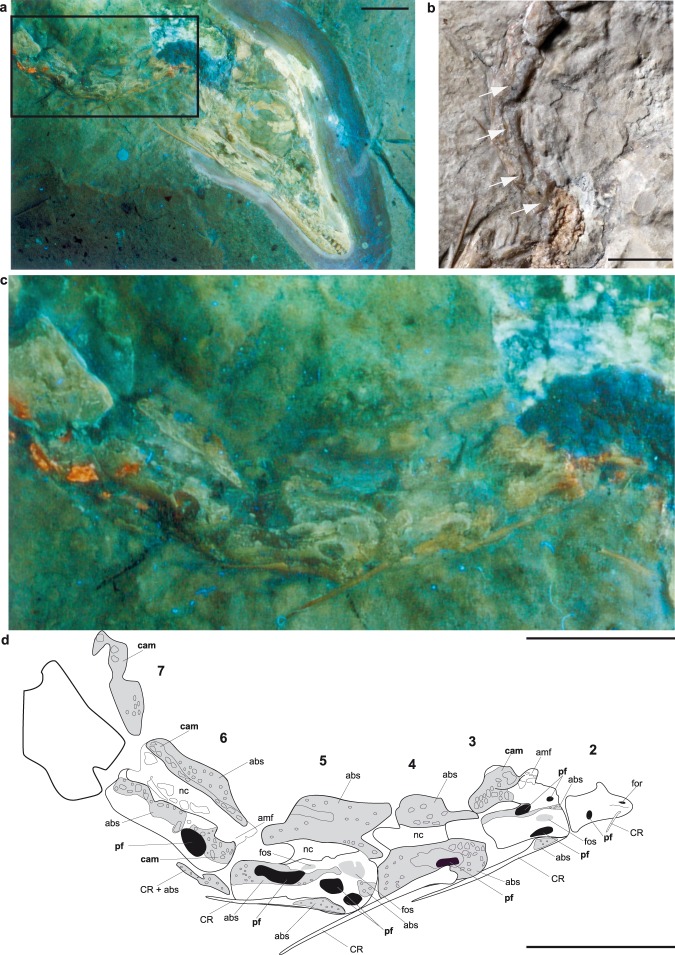
Photographs and interpretative drawing of cervical vertebrae of the Berlin specimen of *Archaeopteryx*, MB.Av.101. (**a**) UV photograph of the neck and skull, (**b**) photograph of the neck under visual light, structures previously described and figured^12^ are marked, (**c**) magnified frame of (**a**) showing details of pneumatic structures in the neck, (**d**) interpretative drawing of (**c**) with recognized pneumatic structures. Anatomical abbreviations used: abs, area of abraded bone surface with regular spongy internal structure; amf, area with unidentified pores or putative camellae; cam, pneumatic camellae; CR, cervical rib; for, foramen; fos, fossa; nc, neural canal; pf, pneumatic foramen. Pneumatic foramina and camellae are unambiguous pneumatic structures and labeled with bold face. Foramina and pneumatic foramina are filled with black; fossae are filled with grey but without margin. Scale bars are 10 mm.

Here, we describe intact and incomplete postcranial bone surfaces in the Berlin *Archaeopteryx* (MB.Av.101; MB = Museum Berlin, Av = Collection of fossil birds), utilizing long-wave UV light^15,18^ to reveal pneumatic structures that are hidden, or difficult to discern, under visible light conditions (Fig. 1). Previous work has shown that UV light is far more sensitive than visible light to the increased contrast between fossilized bone, rocky infill, and surrounding matrix^15,18–21^. A previous description based on UV observations of the Berlin *Archaeopteryx* (MB.Av.101)^15^ has corroborated other reports^1,13^ regarding the presence of pneumatic foramina in the cervical vertebral column and hollow vertebral bodies for the 7^th^ to 10^th^ presacral vertebrae. The new UV findings we present here extend these results, enable a detailed account of all unambiguous pneumatic structures in the postcranial skeleton of MB.Av.101, and confirm that numerous postcranial bones of *Archaeopteryx* were reduced in mass via hollow interiors (Table S1).

## Results

**Figure 2:**
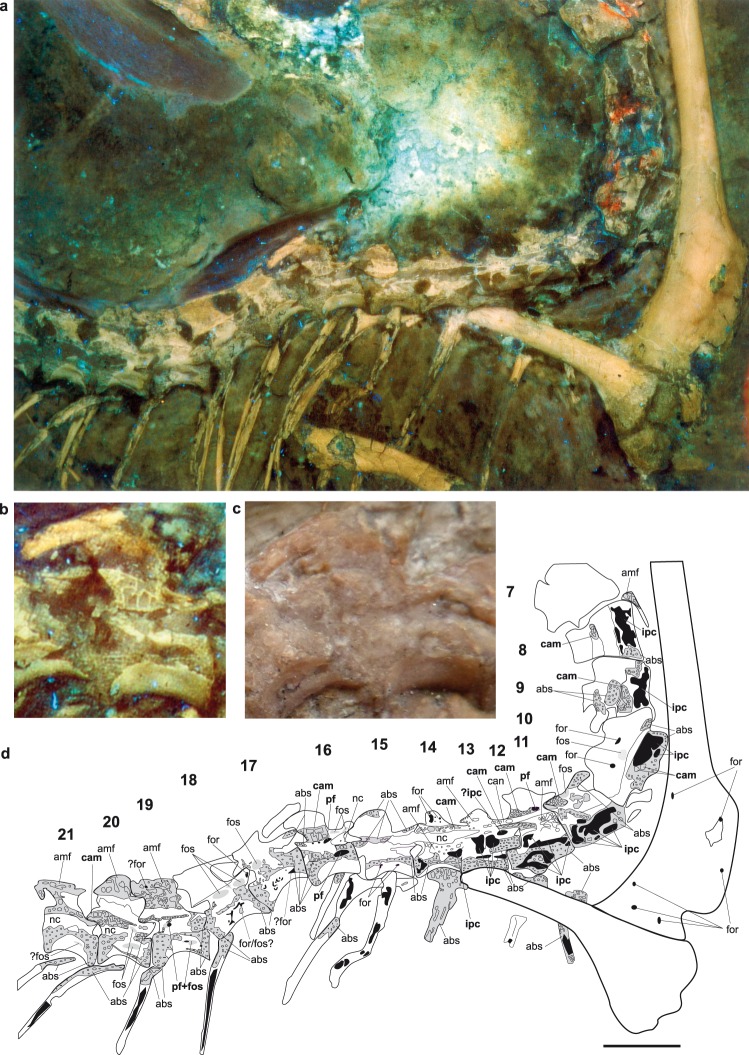
Photographs and interpretative drawing of presacral (thoracic) vertebrae with pneumatic structures of the Berlin specimen of *Archaeopteryx*, MB.Av.101. (**a**) UV photograph, (**b**) magnification of 16^th^ presacral vertebra under UV light, and (**c**) the same vertebra under visible light, (**d**) interpretative drawing of region a. Anatomical abbreviations used: abs, area of abraded bone surface with regular spongy internal structure; amf, area with unidentified pores or putative camellae; cam, pneumatic camellae; for, foramen; fos, fossa; ipc, internal pneumatic camerae; nc, neural canal; pf, pneumatic foramen. Pneumatic foramina, internal camerae and camellae are unambiguous pneumatic structures and labeled with bold face. Foramina and pneumatic foramina are filled with black; fossae are filled with grey but without margin. Scale bars are 10 mm.

**Figure 3:**
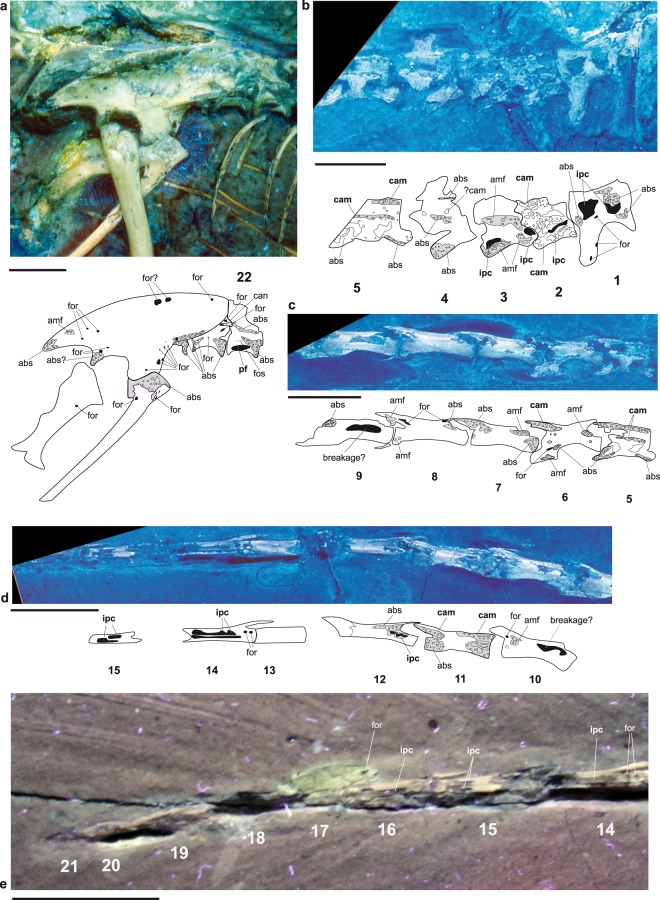
UV photographs and interpretative drawings of the pelvis and sacral and caudal vertebrae with pneumatic structures of the Berlin specimen of *Archaeopteryx*, MB.Av.101. (**a**) Pelvic region in lateral view, with the last (22^nd^) presacral vertebra, (**b**) 1^st^ to 5^th^ caudal vertebra, (**c**) 5^th^ to 9^th^ caudal vertebrae, (**d**) 10^th^ to 15^th^ caudal vertebrae, (**e**) 14^th^ to 21^st^ (last) caudal vertebrae. Anatomical abbreviations and filling of structures as in Figs 1 and 2. Scale bars are 10 mm.

In the Berlin *Archaeopteryx*, the vertebral cortex has been abraded in most presacral vertebrae, possibly due to repeated preparation^15,18^, exposing the internal bone structure of the vertebrae and ribs. UV light conditions expose pneumatic structures more clearly than under visible light, and reveal a far more extensive distribution of intraosseous pneumaticity than has been described since the 19^th^ century. Sharp-lipped pneumatic foramina, which are distinguished from nutrient foramina by their larger diameter^13^, are present at the vertebral body and neural arch of the 2^nd^ to 5^th^ cervical vertebrae, as reported before^1,13,15^. Pneumatic foramina can also be identified on the 6^th^, 16^th^, and 22^nd^ presacral vertebrae (Figs 1 and 2, Table S1). In the 19^th^ presacral vertebra, a combination of a fossa with a communicating foramen is visible at the vertebral body (Fig. 2d). In contrast, small foramina visible in most of the vertebrae likely represent nutrient foramina, and are therefore not evidence for intraosseous pneumaticity^1,13^. In the 8^th^ to 14^th^ presacral vertebrae (Fig. 2), and the 1^st^ to 3^rd^, 12^th^, and 14^th^ to 16^th^ caudal vertebrae (Fig. 3), the interiors exhibit large internal pneumatic chambers, or camerae. The 3^rd^, 8^th^ to 14^th^, 16^th^, and 20^th^ presacral vertebrae, and the 2^nd^, 5^th^, 6^th^ and 11^th^ caudal vertebrae expose pneumatic camellae. These camellae are small, sometimes rounded but mostly angular voids, separated from each other by thin bone walls (Figs 1 and 2b,c); they form a characteristic honeycomb-like pattern that can also be observed in the pneumatic vertebrae of extant birds^1^. In most vertebrae, the camellae are combined with unambiguous pneumatic foramina and large internal camerae (Table S1) typical for pneumatized bones, and are more often preserved in the neural arches than in the vertebral bodies.

At the vertebral bodies, the abraded cortical bone exposes an internal structure that consists of regular, small, and mostly oval pores that are only half as large as the camellae (abs). Rounded and irregularly distributed voids the same size as camellae are visible (amf) in other areas, mostly along the neural arches and in particular all presacral vertebrae and the 3^rd^ and 6^th^ to 8^th^ caudal vertebra (Fig. 1–3). These structures cannot be unambiguously identified as pneumatic structures, as they do not correspond to a typical camellate pattern as described above, but might instead represent areas of spongy bone. The presence of spongy internal bone structure can be taken as evidence for lightweight vertebrae in MB.Av.101. The thoracic ribs also exhibit a pattern comprising small regular and irregular voids in their heads and along their shafts, while their internal shafts are hollow^13^ (Fig. 2a,d; Table S1). A pattern of small and irregular pores can be observed in the posterior process and ventral margin of the ilium, and the head of the pubis (Fig. 3). Only small putative nutrient foramina are observed within the humerus, pelvic bones, femur, and tibia (Figs 2a,d and 3a, Table S1), so unambiguous evidence for intraosseous pneumaticity in the appendicular skeleton is still unknown^1,8^.

The overall skeletal pneumaticity in *Archaeopteryx* was calculated using the Pneumaticity Index (PI), as it provides a quantitative measure for the standardized comparison of postcranial pneumaticity among multiple avian species, independent of phylogeny, body size, and behavior^22^. Mapping of intraosseous pneumaticity in the Berlin *Archaeopteryx* yielded a PI of at minimum 0.39 (7 of 18 anatomical units being pneumatic). This value is comparable to the PI of a few of the extant anseriform birds previously reported, such as *Mergus merganser* and *Anas georgica*^22^ (both 0.41).

**Figure 4:**
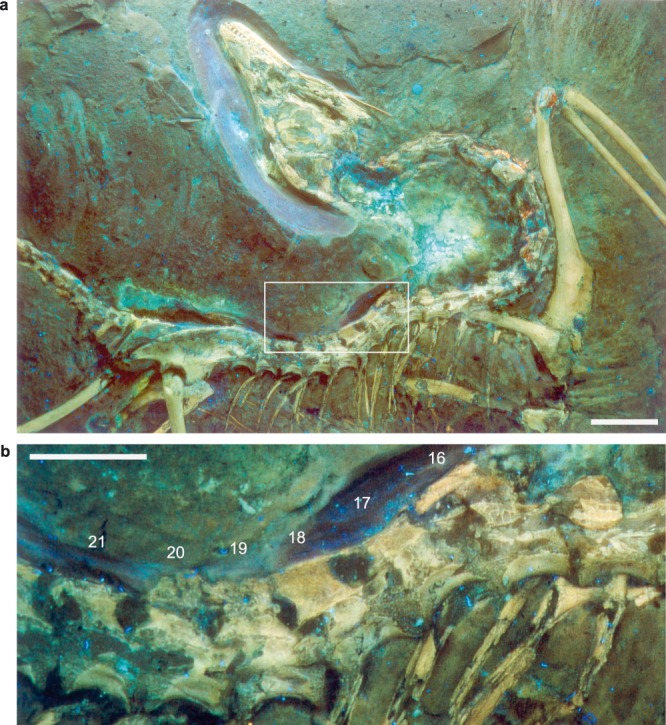
UV photographs of the Berlin specimen of *Archaeopteryx*, MB.Av.101, with magnified region of the fused presacral neural spines forming a notarium-like structure. (**a**) Axial skeleton of *Archaeopteryx*, white frame demarks area of fused neural spines; scale bar is 20 mm. (**b**) Magnification of the 16^th^ to 22^nd^ presacral vertebrae, white arrows mark ossifications between the neural spines and remnants of broken spinal processes; scale bar is 10 mm.

Our UV light observations also corroborate the presence of a notarium-like structure of fused presacral vertebrae in the Berlin *Archaeopteryx*. Fused anterior thoracic vertebrae have been mentioned briefly before^23^, but never described in detail. Our images reveal the presence of a massive, rod-shaped ossification above the apex of the 16^th^ presacral neural spine and connecting to the anterior spine of the 17^th^ (Fig. 4a,b). The caudodorsal corner of the 17th neural spine and the craniodorsal corner of its 18^th^ counterpart comprise a long, thin, rod-like structure that bridges and tightly binds the neural spines together (Fig. 4a,b). The neural spines of the 19^th^ to 20^th^ presacral vertebrae are equally drawn out in their anterodorsal and posterodorsal corners, and fused dorsally with their adjacent neural spines. However, the connection between these elements is partly broken (Fig. 4a,b), while the neural spine of the 21^st^ presacral vertebra has extended anterodorsal and posterodorsal corners.

## Discussion

It is generally accepted that the clearest marker of skeletal pneumaticity is the presence of intraosseous pneumatic structures correlated with foramina^1,7,24^. Hitherto, the presacral distribution of pneumatic structures in *Archaeopteryx* has been discussed solely on the basis of described pneumatic foramina, which is considered controversial^1,7^. Previous workers have noted the existence of such pneumatic foramina on the surfaces of the cervical and thoracic vertebrae, humerus, and pubis of the Berlin, London, Eichstätt, and Thermopolis specimens of *Archaeopteryx*^1,7,8,12–17,25^ (Table S2). However, the most recent integrative study concluded that just the cervical and anteriormost thoracic vertebrae were pneumatized in *Archaeopteryx*, analogous with the plesiomorphic ‘common pattern’ seen in basal theropod dinosaurs^7^. In this study, we expand our knowledge of the Berlin *Archaeopteryx* with the first evidence of unambiguous intraosseous pneumatic structures (i.e., internal cavities such as camerae and camellae). The UV findings now enable convincing identification of internally pneumatic presacral vertebrae (2^nd^ to 14^th^, 16^th^, 20^th^, 22^nd^) and caudal vertebrae (1^st^ to 3^rd^, 5^th^, 6^th^, 11^th^, 12^th^, 14^th^ to 16^th^), demonstrating the extension of pneumatic foramina and intraosseous pneumatic structures to most the vertebral column. Unambiguous traces of vertebral pneumaticity are absent in the remaining thoracic vertebrae, which is consistent with other specimens of *Archaeopteryx*^1,7,13,14,26^. The thoracic ribs are hollow, which has also been reported for the12^th^ specimen of *Archaeopteryx*^26^. The presence of intraosseous pneumaticity in the remaining caudal vertebrae remains unclear, due to the lack of a combination of unambiguous pneumatic structures. We found evidence for at least a spongy and therefore light bone architecture in the 1^st^ and 2^nd^ sacral vertebra, the ilium and the pubis, as well as in some other caudal vertebrae (Fig. 3).

Our survey revealed furthermore that the abraded and broken lumbar vertebra of the Maxberg specimen^27^ also includes a pneumatized internal vertebral body including camellae (Table S2). Additionally, X-ray images of the thoracic vertebrae of the Maxberg *Archaeopteryx*^27^ and the cervicals of the Thermopolis specimen^25^ corroborate the presence of camellae within these vertebrae (Table S2). Additionally, camellate intraosseous pneumatic structures in the last two cervical vertebrae, as well as a pneumatic foramen in the 1^st^ thoracic vertebra (Rauhut *et al*. 2018, p.30; Fig. 17), have been described for the most recently reported 12^th^ specimen of *Archaeopteryx*^26^. Therefore, the presence of intraosseous postcranial pneumaticity in at least four specimens of *Archaeopteryx* including the Berlin, Maxberg, Thermopolis, and the 12^th^ specimen, is documented (Table S2). At least in two specimens, the Maxberg and the Berlin *Archaeopteryx*, pneumatic structures extend outside the region of the anterior thoracic vertebrae. A hollow caudal vertebra is described for the 12^th^ *Archaeopteryx*^26^, although it is unclear whether this represents intraosseous pneumaticity. Clearly seen in the Berlin *Archaeopteryx*, the pattern of intraosseous pneumaticity comprises a combination of closely-spaced honeycomb-shaped camellae bounded by thin bone walls (Fig. 2b), essentially similar to that of extant birds^1,6,8,10^. Camellate pneumatic bone is also found in a range of non-avialan dinosaurs (e.g. *Aerosteon*^28^, *Tyrannosaurus*^29^, and other neotheropods^8^) and in the avialan *Rahonavis*^30^, whereas large internal pneumatic camerae have been less frequently reported (e.g., in megalosauroids^31^, dromaeosaurids^8^ and ornithomimosaurs^32^).

Our new data demonstrate that the pneumaticity status of *Archaeopteryx* does not, in fact, conform to the plesiomorphic “common pattern” of non-avialan theropod dinosaurs^7^. Instead, *Archaeopteryx* possesses what can be termed “extended pattern of pneumaticity” (EPP)^7^, which includes the posterior thoracic vertebrae, and at least some anterior caudal vertebrae. The EPP is also seen in basal Neotheropoda^6,7^, as well as in most neotheropod clades, including Tyrannosauroidea^28^, Oviraptorosauria^33–35^, Dromaeosauridae^36^, and Troodontidae^37,38^, where it is thought to have evolved independently^7^. Among Maniraptora, only Oviraptorosauria possesses a stronger pneumatized vertebral column, including the complete tail^39^. Apart from *Archaeopteryx*, evidence of postcranial pneumaticity within Avialae is patchy, possibly related to the small body size and fragmentary preservation of most specimens. Pneumatic foramina are documented for the cervical vertebrae of confuciusornithid birds^40^, and for the cervical and anterior thoracic vertebrae of *Rahonavis* and some enantiornithid birds^30^, which would conform to a “common pattern” of intraosseous pneumaticity. One report of a pneumatic foramen in the humerus of an enantiornithid bird^41^ might indicate an EPP, but the evolutionary development of this pattern within Avialae still remains unclear. In any case, the level of pneumaticity we found in *Archaeopteryx* already corresponds with the EPP seen in some extant neornithine birds, including non-diving anseriforms^22^.

**Figure 5:**
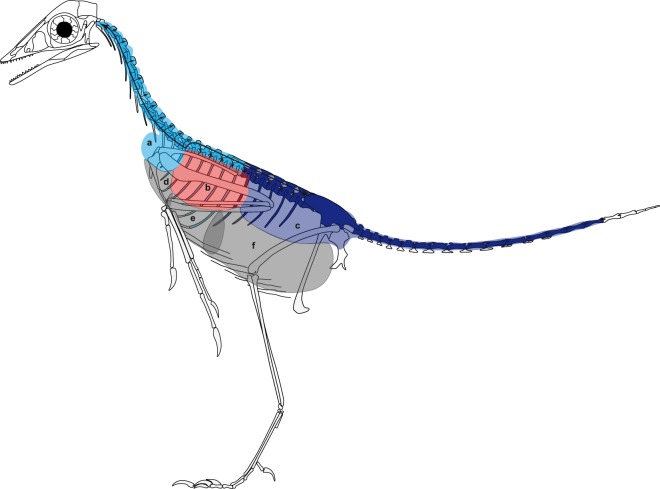
Skeleton of *Archaeopteryx* (Berlin specimen, MB.Av.101) with schematic representation of air sacs and pneumatic postcranial elements as deduced from this study. Skeleton is shown from left lateral side and paired elements are represented only from the left body side. Black arrows indicate the path of pneumatization within air sacs. Light blue = cervical air sac (**a**), red = lung (**b**), dark blue = abdominal air sac (**c**). Grey = clavicular (**d**), anterior thoracic (**e**), and posterior thoracic (**f**) air sacs; these have no osteological evidence in *Archaeopteryx*, making their presence uncertain. Skeleton redrawn from Wellnhofer^26^.

The new evidence of intraosseous pneumatization in *Archaeopteryx* presented here indicates that the respiratory system of this avialan included an air sac distribution similar to that of living birds^1,3,6,22^ (Table S1). The extensive intraosseous postcranial pneumatic structures provide unambiguous evidence for the presence of cervical air sacs that pneumatize cervical vertebrae and ribs, as well as anterior to mid-thoracic vertebrae and thoracic ribs. Intraosseous pneumatization of the mid- to posterior thoracic and caudal vertebrae provide unambiguous evidence for the existence of abdominal air sacs^1,3,22^ (Fig. 5). There is no osteological evidence for the presence of clavicular and anterior thoracic air sacs surrounding the lungs, which would pneumatize the humerus^22^ (Fig. 2, Table S1). The exact position of the lungs also cannot be reconstructed, because this organ is not associated with osteological traces; the lack of pulmonary foramina in thoracic vertebrae of *Archaeopteryx* makes it unlikely that lung tissue pneumatized these vertebrae on a large scale^1^. Similarly, the presence of posterior thoracic air sacs, present in extant birds, cannot be reconstructed for *Archaeopteryx* because these air sacs do not pneumatize the skeleton^1,3,22^. The reconstruction of air sacs in *Archaeopteryx* (Fig. 5) confirms the already assumed^1,7^ presence of a bird-like, high-compliance lung-air sac respiration system, incorporating lungs fixed at the rigid thoracic vertebral column and ventilated by air sacs positioned anteriorly and posteriorly to it.

The rostrad extension of skeletal pneumatization in theropod dinosaurs is a “centrum-first” pattern^7^, which means that first the vertebral bodies are pneumatized via pneumatic foramina (“intramural pneumatization”)^42^, followed by the neural arches. This is consistent with the occurrence of pneumatic foramina throughout the cervical vertebral column of the Berlin specimen (Fig. 1). In the posterior direction, pneumatization proceeds in a “neural arch-first” pattern, with the neural arches being pneumatized first, followed by pneumatization of the vertebral bodies^1,7^. Pneumatic diverticula can proceed through the medullary region first, and enter the vertebral body internally, as in extant birds^1,10,43^. This pattern likely explains the lack of pneumatic foramina observed in the thoracic vertebral bodies in *Archaeopteryx* (Fig. 2), which contrasts its rich intraosseous pneumatic structures. Such an absence of pneumatic foramina in the mid- thoracic region is consistent with many extant birds, in which pneumatic foramina are reduced in the thoracic vertebral series towards the sacrum^1^.

Pneumatization of the postcranial skeleton has a direct weight-reducing effect, by replacing bone and marrow with air-filled cavities^42,44,45^. A correlation between the degree of vertebral pneumaticity and body size was found in large-bodied, non-avian theropod dinosaurs^7^. In extant birds, the degree of postcranial pneumaticity has traditionally been linked to body mass and diving behavior, with the assumption that larger-bodied birds are more pneumatic than smaller-bodied birds, and the known absence of intraosseous pneumaticity in diving birds such as penguins^22^. At least within anseriform birds, no statistically significant relationships between body size and Pneumaticity Index has been found^22^, so that reduced or absent intraosseous pneumaticity (such as in diving taxa) seems not to be generally correlated with the absence of active flight. In smaller extant birds, the threshold of a critical body mass related to the energetic requirements of flight is not reached by a reduction or increase of intraosseous pneumaticity, meaning that the amount, presence, or absence of postcranial intraosseous pneumaticity gives no information on the flight capability of an extant bird^22^. However, if the metabolic activity of a fossil avialan such as *Archaeopteryx* was different than that of extant birds, then the increase of intraosseous pneumaticity might very well have had an effect on the critical body mass, and thereby also the flight capability in *Archaeopteryx*. Nevertheless, given the remaining uncertainty in the reconstruction of both the total extension of postcranial intraosseous pneumaticity and the presence of all pulmonary air-sacs, the precise estimation of body mass in *Archaeopteryx* remains speculative. A hypothetical adult (somatically mature) *Archaeopteryx* has been reconstructed to exceed 500 mm in body length and have a body mass between 0.8 and 1 kg, comparable to a raven (*Corvus corax*)^46^, values which we assume to be within the possible ranges for this taxon and not contradicted by the new data on intraosseous pneumaticity in this study.

The documentation of stiffening structures in neural spines of the thoracic vertebral column of *Archaeopteryx* is rather novel, and we argue that these ossifications represent the earliest known occurrence of a notarium-like structure on the line to extant birds, pushing back its origin to Paraves. Previously, spinal processes in the thoracic vertebrae had only been described in late Cretaceous enantiornithine birds^41^. It is also interesting to note the absence of stabilization structures in basal birds more derived than *Archaeopteryx*, such as the Confuciusornithidae, as well as the heterogeneous distribution of notaria in extant birds^47–51^. The apparent absence of these structures in other specimens of *Archaeopteryx* must remain speculative, and might be due to differing degrees of tendon ossification and/or preservation in these specimens. In several members of extant avian groups such as Tinamiformes, Podicipediformes, Phoenicopteriformes, Galliformes, Columbiformes, Pelecaniformes, Falconiformes, Gruiformes, and Caprimulgiformes, the vertebrae of the thorax form a notarium; this is a rigid structure created by 2–5 ankylosed vertebrae, including ossified tendons and ligaments, and separated in most cases from the synsacrum by at least one “free” or unankylosed vertebra^47–51^. Importantly, the fusion of neural spines in *Archaeopteryx* lies in the thoracic vertebral column between the 16^th^ and the 22^nd^ presacral vertebrae (Fig. 4a,b), corresponding to the notarium region in extant avians^51^, and there is also a vertebral gap between the fused and sacral regions. The narrow, ‘rod-like structures along the neural spines are interpreted to represent ossified ligaments^52^ or tendons of the epaxial trunk musculature. These would have acted as bony splints^52^, and likely contributed to an incipient ankylosis between the vertebrae (although the vertebral bodies themselves are not fused with each other).

A relatively rigid thoracic vertebral column is required for a bird-like respiration mechanism^3^, and is therefore an important structure to be documented in *Archaeopteryx*. In extant birds, the biological importance of the notarium is explained mostly as a rigid stabilization structure, counteracting mechanical stress occurring during wing-driven flapping flight and acting as a shock absorber during landing^50^. A notarium has also been described for some pterosaurs as an adaptation to active flight^53,54^. In any case, fusion of the neural spines creates a more stable (but less flexible) region in the vertebral column, which acts as a unit and can be more effectively stabilized by the available muscles and ligaments against the mechanical forces during locomotion.

Ultimately, reinforcement structures in the thoracic vertebral column can be interpreted to facilitate novel locomotor modes, in particular the development of active flapping flight or bipedal running supported by flapping wings, that increases stresses acting on the vertebral column. There has been controversial anatomical evidence on the ability of *Archaeopteryx* for gliding or flapping flight^27,55–59^. Nevertheless, the reinforcement structures observed in the trunk is indirect evidence for the increased use of forelimbs in *Archaeopteryx*, and depicts a stabilization trend that continues on the line to extant birds with the reduction in the number and increasing intervertebral fusion of thoracic vertebrae into a regional notarium^47,48,52^.

## Conclusions

We demonstrate that *Archaeopteryx* possessed a derived, bird-like postcranial pneumatization pattern that comprises intraosseous pneumatic camellae and camerae within the presacral vertebral column and caudal vertebrae. The new observations furthermore confirm that expanded postcranial pneumatic structures and a bird-like respiratory system, both important physiological adaptations for powered flight, were present in *Archaeopteryx*. Whereas the number of pneumatic bones has a direct effect on weight reduction in large-bodied dinosaurs^5,7^, the camerate and camellate architecture of pneumatized bone is more likely determined by mechanical factors in the vertebral column, in concert with the evolutionary development and phylogenetic integration of the taxa. In *Archaeopteryx*, the direct effect on weight-reduction of the vertebral column by intraosseous pneumatic structures was moderate. As an indirect effect, the lightened pneumatic vertebral column would have needed less muscle forces to be stabilized against the mechanical loads that occur during all locomotor modes. Intraosseous pneumatization in *Archaeopteryx* was important for the metabolism of the animal, because the pneumatic epithelium replaces metabolically costly and massive bone, which reduces metabolic energy consumption and locomotion costs, and helped to increase the metabolic performance of the animal^7,45,59,60^. The putative high energetic advantage of a pneumatic postcranium^7,24^ demonstrates the particular importance for a correct establishment of the pneumaticity status in *Archaeopteryx*. The presence of an expanded pneumatization pattern in *Archaeopteryx* indicates facilitation of an active lifestyle^7,12^ and allows characterizing *Archaeopteryx* as a taxon that already had the metabolic prerequisites for this highly demanding active lifestyle and an avian-like, high-performance endothermy. The presence of a notarium-like stabilizing structure in the vertebral column in *Archaeopteryx* is fully in line with the new evidence on its expanded pneumaticity pattern, and adds another piece of evidence for the potential of this taxon for active, wing-driven flapping flight.

## Methods

The main slab of the Berlin specimen of *Archaeopteryx*, MB.Av.101, was observed directly (by the naked eye) and with microscopy. The images taken by Helmut Tischlinger with the help of longwave UV light (365–366 nm) in combination with selective filter techniques^15,21^ were examined carefully and compared to the original specimen. The UV photographs are available at the MfN. Additionally, new photographs under longwave UV radiation were taken by M.K. to supplement the older images and compare both of them. Comparative photographs under visible light were provided by R.C.

A survey of other described specimens of *Archaeopteryx* has been done mainly by literature to get an idea of potentially preserved pneumatic structures. The Maxberg specimen of *Archaeopteryx* is lost and therefore could only be examined by photographs from Wellnhofer^27^. Data on the Thermopolis specimen of *Archaeopteryx* were taken from Mayr *et al*.^25^.

We assume that the first preserved vertebra visible in the head region represents the 2^nd^ cervical because of its integration with the occiput; thus, the first vertebra with a thoracic rib is the 11^th^ presacral vertebra (which has its rib slightly caudoventrally emplaced beneath the scapula), and so the first sacral vertebra (the rostralmost vertebra without a thoracic rib) is medial to the anterior iliac blade. We count therefore 22 presacral vertebrae in this specimen (i.e., 10 cervicals, and 12 rib-bearing thoracic vertebrae), one less than in the other known specimens of *Archaeopteryx*^14,27^.

We calculated a Pneumaticity Index^22^ (PI) as a quantitative measure to compare the amount of pneumatic elements throughout the postcranial skeleton with some extant birds. The PI is defined as the ratio between the number of pneumatic postcranial elements and the total number of postcranial elements^22^. Postcranial elements are subdivided into anatomical units (AU), yielding the equation of PI = #AU pneumatic/#AU total. The following AUs (see also Table S3) were defined for *Archaeopteryx:* Composite units = **ANC**, Anterior Cervical Vertebrae (1^st^ to 3^th^ cervical); **MC**, Middle Cervical Vertebrae (4^th^ to 6^th^ cervical); **POC**, Posterior Cervical Vertebrae (7^th^ to 10^th^ cervical); **ANT**, Anterior Thoracic Vertebra (1^st^ to 6^th^ thoracic); **POT**, Posterior Thoracic Vertebrae (7^th^ to 12^th^ thoracic); **SS**, Synsacral Vertebrae; **CAA**, Anterior Caudal Vertebrae; **CAP**, Posterior Caudal Vertebrae; **TR**, Thoracic Ribs; **CAR**, Caudal Ribs and Chevrons; **PE**, Pelvis (Ilium, Ischium, Pubis); **DFL**, distal forelimb elements (i.e., bones distal to elbow joints); **DHL**, distal hind limb elements (i.e., bones distal to knee joints); Individually scored units = **CC**, coracoids; **FU**, furculae; **FM**, femora; **HU**, humeri; **SC**, scapulae. In the Berlin specimen of *Archaeopteryx*, 7 (ANC, MC, POC, ANT, POT, CAA, TR) of the listed 18 AUs are unambiguously pneumatic, yielding a minimum **PI of 7:18 = 0.39**.

## Supplementary information

Supplemental Information

## Data Availability

The original Berlin specimen of *Archaeopteryx*, MB.Av.101, is housed in the collection of fossil vertebrates at the Museum für Naturkunde in Berlin (MfN) and can be studied upon request at the institution. The authors declare that the data supporting the findings of this study are available within the paper and its supplementary information files.
