# Supplementary material for: Ultraviolet light illuminates the avian nature of the Berlin Archaeopteryx skeleton
Source: Sci Rep. 2019 Apr 24;9:6518. doi: 10.1038/s41598-019-42823-5 (PMC6482141; doi:10.1038/s41598-019-42823-5)
Supplement: Supplementary file 1 — Supplemental Information [file 41598_2019_42823_MOESM1_ESM.pdf]

Supplemental Information to

Ultraviolet light illuminates the avian nature of the Berlin *Archaeopteryx* skeleton

Daniela Schwarz[1*], Martin Kundrát[2*], Helmut Tischlinger[3], Gareth Dyke[2,4], Ryan M. Carney[5]

[1] Museum für Naturkunde, Leibniz Institute for Evolution and Biodiversity Science, 9115 Berlin, Germany.

[2] Center for Interdisciplinary Biosciences, Technology and Innovation Park, University of Pavol Jozef Šafárik, 04154 Košice, Slovakia.

[3]Tannenweg 16, 85134 Stammham, Germany.

[4] Department of Geology, Babes-Bolyai University, Romania.

[5] Department of Integrative Biology, University of South Florida, 33620 Tampa, FL, USA.

**Vertebral Position**	**Unambiguous pneumatic structures: pneumatic foramina (pf), internal pneumatic camerae (ipc), camellae (cam)**	**Ambiguous pneumatic structures: small pores of similar size and regular distribution(abs), irregularly distributed small rounded putative camellae (amf)**	**Putative nutrient foramina**	**Pneumatizing air sac[1]**
Presacral 2, Axis (Fig. 1)	high oval pf posteriorly at lateral face vertebral body	none	small foramen at lamina of prezygapophysis	cervical air sac
Presacral 3, 3rd Cervical (Fig. 1)	long oval pf ventrally at lateral face of vertebral body, pf at lateral face of vertebral body and pf ventral to praezygapophysis, rounded rectangular camellae at neural arch and neural spine	fossa in dorsal half of lateral vertebral body; abraded lateral surface of vertebral body reveals abs; amf along lateral face of prezygapophysis; bone abrasion reveals abs at head of cervical rib	none	cervical air sac
Presacral 4, 4th	long oval pf at lateral face of vertebral body	abraded bone exposes pattern of abs filling	none	cervical air sac

Cervical, (Fig. 1)		vertebral body and neural arch and spine		
Presacral 5, 5th Cervical (Fig. 1)	two rounded pf in rostral half of lateral face of vertebra body, one pf in posterior half of lateral face of vertebral body	two fossae visible laterally at vertebral body; abs visible at lateral vertebral body anteriorly and around pneumatic foramen and at neural spine	none	cervical air sac
Presacral 6, 6th Cervical (Fig.1)	long oval pf at lateral face of vertebral body, rounded angular camellae at lateral face of vertebral body and base of neural arch along a line connecting pre- and postzygapophysis	neural canal shows a pattern of rounded rectangular voids that might be camellae; abs at abraded lateral surface of neural spine and at cervical rib head with spongy bone	none	cervical air sac
Presacral 7, 7th Cervical (Fig. 1)	fragment of neural arch reveals camellae	problematic bone fragment at vertebral body that might have been added during preparation[2], not included into observation here	none	cervical air sac
Presacral 8, 8th Cervical (Fig. 2)	interior of vertebral body hollowed out by ipc in its central two-thirds; camellae visible at lateral neural arch	abraded lateral surface in posterior half of vertebral body with abs, cervical rib head with amf		cervical air sac
Presacral 9, 9th cervical (Fig. 2)	interior of vertebral body hollowed out by ipc in its central two-thirds; abraded bone in posterior part of lateral vertebral body exposes camellae	Abraded lateral surface of vertebral body (anteriorly) and neural arch (posteriorly) exposes abs	none	cervical air sac
Presacral 10, 10th cervical (Fig. 2)	interior of vertebral body hollowed out by ipc in its anterior half; another pneumatic cavity ventrally; 2 unambigous camellae at lateral face of vertebral body posterior to ipc	Abraded lateral surface of vertebral body exposes abs around ipc; fossa laterally at neural arch	Lateral face of neural arch with two foramina	cervical air sac
Presacral 11, 1st thoracic (Fig. 2)	interior of vertebral body hollowed out by ipc in its central two-thirds; ipc ventral to diapophysis; camellae on neural arch dorsal to diapohysis	Abraded lateral surface of vertebral body, diapophysis and lateral face of prezygapophysis exposes abs, oval fossa at neural arch between diapophysis and prezygapophysis	none	cervical air sac
Presacral 12, 2nd	interior of vertebral body hollowed out by ipc in its central two-thirds; ipc	abraded cortical bone on lateral vertebral body exposes abs; canal like	none	cervical air sac or pulmonary

thoracic (Fig. 2)	ventral to diapophysis; dorsal face of diapophysis with abraded cortical bone exposing camellae and combined with pf with aperture in lateral neural spine, camellae continue posteriorly along the prezygapophysis of the 13th presacral vertebra	hollow area at base of postzygapophysis; neural arch dorsal to postzygapophysis laterally abraded exposing spongy internal bone structure; rostral part of lateral neural arch exposes amf		diverticula of lung
Presacral 13, 3rd thoracic (Fig. 2)	vertebral body medially with 2 narrow and elongate ipc, anterior and posterior to diapophysis each a large ipc, rounded pf or ipc ventral to prezygapophysis; large pneumatic cavities under diapophysis	ventral two-thirds of lateral vertebral body and anterior part of neural arch base and vertebral body with abs; lateral face of neural spine exposes amf	none	cervical air sac or pulmonary diverticula of lung
Presacral 14, 4th thoracic (Fig. 2)	ipc ventral at prezygapophysis; camellae at base of neural spine along a lamina from pre- to postzygapophysis	Dorsal half of lateral vertebral body with abs and deep preservational cavity; surface of diapophysis and base of neural arch ventral to neural canal covered with pattern of amf; lateral face of neural spine exposes large opening that might be either pneumatic foramen or preservational	Lateral face of neural spine with a number of small circular foramina	cervical air sac or pulmonary diverticula of lung
Presacral 15 (Fig. 2)	none	dorsal half of lateral vertebral body laterally with abs; most of neural arch is crushed; abs visible at prezygapophysis, base of neural spine and apical region of neural spine	two small foramina in ventral half of lateral face of vertebral body	cervical air sac or pulmonary diverticula of lung
Presacral 16 (Fig. 2)	In anterior half of lateral face of vertebral body drop shaped and sharply bounded pf; long-oval pf medially at lateral face of neural arch; rectangular camellae hollow out postygapophysis	Lateral face of vertebral body and region ventral to postzygapophysis exposes abs with regionally larger hollows that might be camellae; two fossae laterally at prezygapophysis	small foramina lie within neural canal and at base of neural arch posterior to pneumatic foramen	cervical air sac or pulmonary diverticula of lung
Presacral 17 (Fig. 2)	none	remnant of possible foramen visible at anterior part of lateral	none	Probably abdominal air sac

		vertebral body; lateral surface of vertebral body, base of neural arch and lateral surface of prezygapophysis with abs; lateral surface of vertebral body medially with small, irregular foramina that might be preservation-related resorption traces; large bowl-shaped fossa visible ventrally to postzygapophysis at caudolateral part of neural arch		
Presacral 18 (Fig. 2)	none	oblique and oval fossa or foramen at lateral face of vertebral body; distinct long-oval fossa at lateral face of neural arch base, anteriorly followed by a narrow and triangular fossa, and a deep long oval fossa at lateroventral face of prezgyapophysis; irregular foramina that might be preservation-related resorption traces visible at vertebral body; abraded surface exposes abs at lateral face of vertebral body	foramen anteriorly at prezygapophysis	abdominal air sac
Presacral 19 (Fig. 2)	Combination of elongate fossa with a narrow pf in anterior half of lateral vertebral body and at base of neural arch ventral to postzygapophysis	Lateral surface of vertebral body and base of neural arch with abs; lateral surface of neural spine eroded and exposes amf	putative foramen (or result of erosion of bone) visible laterally at neural spine	abdominal air sac
Presacral 20 (Fig. 2)	Abraded bone surface exposes camellate internal bone structure at lateral side of postzygapophysis and neural arch base	Elongate fossa visible at lateral face of vertebral body, obliquely oriented oval shaped fossa at lateral side of neural arch; lateral side of vertebral body exposes anteriorly and posteriorly abs; neural arch at base along prezygapophysis and along lateral face of neural spine with amf	none	abdominal air sac

Presacral 21 (Fig. 2)	none	small, elongate, slitlike putative fossa in anterior third of lateral vertebral body; lateral face of vertebral body exposes abs; amf exposed at lateral face of neural spine	none	abdominal air sac
Presacral 22 (Fig. 3A)	large, elongate sharp lipped pf in posterior two-thirds of vertebral body	fossa dorsal to pf at lateral face of vertebral body; abs visible in anterior part of lateral face of vertebral body and along an area at neural arch connecting pre- and postzygapophysis		abdominal air sac
Thoracic ribs (Fig. 2)	none	abs exposed at heads and along shaft of ribs on 12th, 13th, 14th, 15th, 16th, 18th, 19th, 20th and 21st presacral vertebrae, all thoracic ribs are broken and expose only thin bone walls with strongly hollowed out shafts	none	abdominal air sac
Sacral vertebra 1 (Fig. 3A)	none	abs anteriorly and posteriorly at lateral face of vertebral body, canal like structure with foramina at anterior part of base of neural arch	foramen at medial face of vertebral body, 2 foramina at anterior half of neural arch around prezygapophysis	abdominal air sac
Sacral vertebra 2 (Fig. 3A)	none	small part of vertebral body visible, abraded bone exposes abs	none	abdominal air sac
Caudal vertebra 1 (Fig. 3A)	large irregular ipc in anterior and posterior half of vertebral body	abs is exposed in anterior half of lateral vertebral body and at base of neural arch	foramen at ventral part of neural arch, two foramina at anterior margin of haemapophysis	abdominal air sac
Caudal vertebra 2 (Fig. 3A)	elongate ipc laterally at vertebral body, vertebral body and neural arch filled with camellae	none	none	abdominal air sac
Caudal vertebra 3 (Fig. 3A)	rounded ipc in anterior and posterior half of lateral vertebral body	lateral face of vertebral body and base of neural arch expose amf	none	abdominal air sac

Caudal vertebra 4 (Fig. 3A)	none	Posterior third of vertebral body, and lateral face of neural arch with abs; putative camellae at base of neural arch	none	abdominal air sac
Caudal vertebra 5 (Fig. 3A, 3C)	camellae at base of neural spine	area with abs at vertebral body and haemapophysis	none	abdominal air sac
Caudal vertebra 6 (Fig. 3C)	camellae along postzygapophysis	abs and amf anteriorly and posteriorly visible at vertebral body and neural arch	foramen in caudal part of lateral vertebral body	abdominal air sac
Caudal vertebra 7 (Fig. 3C)	none	abs and amf at lateral vertebral body and neural arch	none	abdominal air sac
Caudal vertebra 8 (Fig. 3C)	none	small areas with amf and abs are visible at lateral vertebral surface	narrow and slit-like foramina at base of neural arch	abdominal air sac
Caudal vertebra 9 (Fig. 3C)	none	abs at base of neural spine, large sharp lipped foramen occupies medial part of lateral vertebral centrum - this is plausibly interpreted to breakage structure	none	abdominal air sac
Caudal vertebra 10 (Fig. 3D)	none	amf at base of neural spine, large sharp lipped foramen occupies anterodorsal part of lateral vertebral centrum - this is plausibly interpreted to breakage structure	foramen at base of postzygapophysis	abdominal air sac
Caudal vertebra 11 (Fig. 3D)	camellae are visible in anterior half of vertebral body and embedded in pattern of abs, camellae at postzygapophysis	lateral face of vertebral body exposes abs	none	abdominal air sac
Caudal vertebra 12 (Fig 3D)	small rounded and internconnected ipc are visible laterally at vertebral body	lateral face of neural arch exposes abs	none	abdominal air sac
Caudal vertebra 13 (Fig. 3D)	None	none	none	abdominal air sac
Caudal vertebra 14 (Fig. 3D, E)	vertebral body is internally hollowed out by 2 elongate cavities interpreted as ipc	none	two foramina at base of neural arch	abdominal air sac

Caudal vertebra 15 (Fig. 3D, E)	vertebral body is internally hollowed out by 2 elongate cavities interpreted as ipc	none	none	abdominal air sac
Caudal vertebra 16 (Fig. 3E)	vertebral body is internally hollowed out by narrow ipc	none	none	abdominal air sac
Caudal vertebra 17 (Fig. 3E)	none	none	foramen anteriorly at lateral face of neural spine	abdominal air sac
Caudal vertebra 18-20 (Fig. 3E)	none	none	none	abdominal air sac
Humerus (Fig. 2)	None	none	proximal extremity bears five foramina distributed along margins, dorsal part of deltopectoral crest with large break at rounded foramen	clavicular and cranial thoracic air sac
Ilium (Fig. 3A)	none	posterior iliac process with laterally abraded bone exposing abs, small area with amf slightly rostrally to abs at posterior iliac process; narrow zone of abs cranioventrally at iliac margin	Several foramina are positioned along posterior half of iliac blade and pubic process, dorsal margin bears two putative foramina	abdominal air sac
Pubis (Fig. 3A)	none	abs exposed at pubic head	small foramina posteriorly at pubic head and along anterior margin of proximal shaft	abdominal air sac
Ischium (Fig. 3A)	none	none	foramen at ischium shaft	abdominal air sac
Femur	none	none	medial foramen on distal shaft and at mid-shaft height	abdominal air sac
Tibia	none	none	foramina at mid-shaft height	abdominal air sac

Table S1. Details of pneumatic structures observed in the postcranial skeleton of the Berlin specimen of *Archaeopteryx*, MB.Av.101, with references to figures. The presacral vertebral count of the Berlin specimen is taken in accordance with Britt et al.[3]

Specimen	Femur length	Observed pneumatic features
Eichstätt specimen	37.0 mm	Ambiguous: cervical vertebra with laminae and fossae, possibly pneumatic foramen in anterior thoracic vertebra[3].
Munich specimen	48.0 mm	Ambiguous: cervical vertebrae preserve some laminae, mention of a pneumatic foramen in a posterior thoracic by Elzanowski[4].
Thermopolis specimen	50.3 mm[5]	Unambiguous: X-ray figure in Mayr et al.[5] shows camellae within cervical vertebrae.
Berlin specimen	52.6 mm	Unambiguous: UV images show continuous pattern of pneumatic camellae throughout the whole vertebral column and pelvis. Pneumatic foramina in cervical vertebrae as documented by Britt et al.[3] and O'Connor[6]
12th specimen	~53 mm[7]	Unambiguous: intraosseous pneumatic structure in mid-cervical vertebra and 8th cervical vertebra, pneumatic foramen in 1st thoracic vertebra[7]
11th specimen	55.3 mm[8]	Ambiguous: cervical and anterior thoracic vertebrae seem to show laminae and fossae[8].
Maxberg specimen	56.4 mm	X-ray photographs and figure of lumbar and anterior thoracic vertebra in Wellnhofer[2] exposes internal pneumaticity of anterior thoracic and posterior thoracic vertebrae (with hiatus between), centered around neural arch.
London specimen	61.0 mm	Ambiguous: pneumatic foramen in pubis and anterior thoracic vertebral centrum reported by

		Christiansen and Bonde[9], earliest report of camellate pneumatic bone by Owen[10]
Solnhofen specimen	67.0 mm	Poor preservation[2] prevents evaluation of pneumatization status.

Table S2. List of *Archaeopteryx* specimens with preservation of vertebral pneumaticity. Femur lengths are taken from Wellnhofer[2] unless otherwise noted.

O'Connor 2004[1] (n=17)	O'Connor 2009[11] (n=12)	This study (n=18)
Composite units		
**CRC**, Cranial Cervical Vertebrae*<br>**MC**, Middle Cervical Vertebrae*<br>**CAC**, Caudal Cervical Vertebrae*	**CeV**, cervical vertebrae	**ANC**, Anterior Cervical Vertebrae<br>**MC**, Middle Cervical Vertebrae<br>**POC**, Posterior Cervical Vertebrae
**CRT**, Cranial Thoracic Vertebrae<br>**CAT**, Caudal Thoracic Vertebrae	**TV**, thoracic vertebrae	**ANT**, Anterior Thoracic Vertebrae<br>**POT**, Posterior Thoracic Vertebrae
**SS**, Synsacral Vertebrae	**SV**, sacral vertebrae	**SS**, Synsacral Vertebrae
**CA**, Caudal Vertebrae	**CaV**, caudal vertebrae	**CAA**, Anterior Caudal Vertebrae<br>**CAP**, Posterior Caudal Vertebrae
**VR**, Vertebral Ribs<br>**SR**, Sternal Ribs	**CO**, costal elements	**TR**, Thoracic Ribs<br>N/A<br>**CAR**, Caudal Ribs and Chevrons
**CX**, Fused Ilium-Ischium-Pubis	**Plv**, pelvic girdle elements	**PE**, Pelvis (Ilium, Ischium, Pubis)
**DLE**, Distal Limb Segments**	**DFL**, distal forelimb elements<br>**DHL**, distal hind limb elements	**DFL**, distal forelimb elements**<br>**DHL**, distal hind limb elements**
Individually scored units		
**CC**, coracoids<br>**SC**, scapulae<br>**FU**, furculae	**Pct**, pectoral girdle elements	**CC**, coracoids<br>**SC**, scapulae<br>**FU**, furculae
**ST**, sterna	**ST**, sternum	N/A
**HU**, humeri	**Hu**, humerus	**HU**, humeri
**FM**, femora	**Fe**, femur	**FM**, femora

*Cervical ribs were scored with their respective vertebrae, as they are fused in extant birds.
** Distal Limb Elements = bones distal to elbow or knee joints

Table S3. Differences between the Anatomical Units (AUs) and acronyms used in this study and by O`Connor[1,11].

Supplementary references

1. M. P. O'Connor. Pulmonary pneumaticity in the postcranial skeleton of extant aves: a case study examining Anseriformes. *J. Morphol.* **261**, 141 (2004).

2. P. Wellnhofer. *Archaeopteryx. Der Urvogel von Solnhofen*. (Verlag Dr. Friedrich Pfeil, München, 2008), pp. 256.

3. B. B. Britt, P. J. Makovicky, J. Gauthier, & N. Bonde. Postcranial pneumatization in Archaeopteryx. *Nature* **395**, 374 (1998).

4. A. Elzanowski. in *Mesozoic Birds: Above the Heads of Dinosaurs,* L. Chiappe, L. M. Witmer, Eds. (University of California Press, 2002), pp. 129-159.

5. G. Mayr, B. Pohl, S. Hartman, & D. S. Peters. The tenth skeletal specimen of *Archaeopteryx*. *Zool. J. Linn. Soc.* **149**, 97 (2007).

6. M. P. O'Connor. Postcranial pneumaticity: An evaluation of soft-tissue influences on the postcranial skeleton and the reconstruction of pulmonary anatomy in archosaurs. *J. Morphol.* **267**, 1199 (2006).

7. O. W. M. Rauhut, C. Foth, & H. Tischlinger. The oldest *Archaeopteryx* (Theropoda: Avialae): a new specimen from the Kimmeridgian/Tithonian boundary of Schamhaupten, Bavaria. *PeerJ* **6:e4191** (2018).

8. C. Foth, H. Tischlinger, & O. W. M. Rauhut. New specimen of *Archaeopteryx* provides insights into the evolution of pennaceous feathers. *Nature* **511**, 79 (2014).

9. P. Christiansen & N. Bonde. Axial and appendicular pneumaticity in *Archaeopteryx*. *Proc. Roy. Soc. Lond., Ser. B* **267**, 2501 (2000).

10. R. Owen. On the *Archaeopteryx* of von Meyer, with a description of a new long tailed species from the lithographic stone of Solenhofen. Philosophical Transactions of the Royal Society of London **153**, 33 (1864).

11. M. P. O'Connor. Evolution of Archosaurian body plans: skeletal adaptations of an air-sac-based breathing appartus in birds and other archosaurs. *J. Exp. Zool.* **311A**, 629 (2009).
